# The Advantage of Targeted Next-Generation Sequencing over qPCR in Testing for Druggable *EGFR* Variants in Non-Small-Cell Lung Cancer

**DOI:** 10.3390/ijms25147908

**Published:** 2024-07-19

**Authors:** Adam Szpechcinski, Joanna Moes-Sosnowska, Paulina Skronska, Urszula Lechowicz, Magdalena Pelc, Malgorzata Szolkowska, Piotr Rudzinski, Emil Wojda, Krystyna Maszkowska-Kopij, Renata Langfort, Tadeusz Orlowski, Pawel Sliwinski, Mateusz Polaczek, Joanna Chorostowska-Wynimko

**Affiliations:** 1 Department of Genetics and Clinical Immunology, The Institute of Tuberculosis and Lung Diseases, 01-138 Warsaw, Poland; j.moes@igichp.edu.pl (J.M.-S.); jaguspaulina@gmail.com (P.S.); u.lechowicz@igichp.edu.pl (U.L.); m.pelc@igichp.edu.pl (M.P.); j.chorostowska@gmail.com (J.C.-W.); 2 Department of Pathology, The Institute of Tuberculosis and Lung Diseases, 01-138 Warsaw, Poland; m.szolkowska@gmail.com (M.S.); r.langfort@igichp.edu.pl (R.L.); 3 Clinics of Thoracic Surgery, The Institute of Tuberculosis and Lung Diseases, 01-138 Warsaw, Poland; p.rudzinski@igichp.edu.pl (P.R.); t.orlowski@igichp.edu.pl (T.O.); 4 III Department of Lung Diseases and Oncology, The Institute of Tuberculosis and Lung Diseases, 01-138 Warsaw, Poland; emilwojda@wp.pl (E.W.); m.polaczek@igichp.edu.pl (M.P.); 5 II Department of Lung Diseases, The Institute of Tuberculosis and Lung Diseases, 01-138 Warsaw, Poland; p.sliwinski@igichp.edu.pl; 6 Outpatient Clinic, The Institute of Tuberculosis and Lung Diseases, 01-138 Warsaw, Poland; krystynakopij@gmail.com

**Keywords:** non-small-cell lung cancer, molecular diagnostics, epidermal growth factor receptor, mutations, targeted next-generation sequencing

## Abstract

The emergence of targeted therapies in non-small-cell lung cancer (NSCLC), including inhibitors of epidermal growth factor receptor (EGFR) tyrosine kinase, has increased the need for robust companion diagnostic tests. Nowadays, detection of actionable variants in exons 18–21 of the *EGFR* gene by qPCR and direct DNA sequencing is often replaced by next-generation sequencing (NGS). In this study, we evaluated the diagnostic usefulness of targeted NGS for druggable *EGFR* variants testing in clinical NSCLC material previously analyzed by the IVD-certified qPCR test with respect to DNA reference material. We tested 59 NSCLC tissue and cytology specimens for *EGFR* variants using the NGS ‘TruSight Tumor 15’ assay (Illumina) and the qPCR ‘cobas EGFR mutation test v2’ (Roche Diagnostics). The sensitivity and specificity of targeted NGS assay were evaluated using the biosynthetic and biological DNA reference material with known allelic frequencies (VAF) of *EGFR* variants. NGS demonstrated a sufficient lower detection limit for diagnostic applications (VAF < 5%) in DNA reference material; all *EGFR* variants were correctly identified. NGS showed high repeatability of VAF assessment between runs (CV% from 0.02 to 3.98). In clinical material, the overall concordance between NGS and qPCR was 76.14% (Cohen’s Kappa = 0.5933). The majority of discordant results concerned false-positive detection of *EGFR* exon 20 insertions by qPCR. A total of 9 out of 59 (15%) clinical samples showed discordant results for one or more *EGFR* variants in both assays. Additionally, we observed *TP53* to be a frequently co-mutated gene in *EGFR*-positive NSCLC patients. In conclusion, targeted NGS showed a number of superior features over qPCR in *EGFR* variant detection (exact identification of variants, calculation of allelic frequency, high analytical sensitivity), which might enhance the basic diagnostic report.

## 1. Introduction

Currently, available targeted therapies in lung cancer are dedicated to patients with tumors exhibiting features of oncogene addiction. Most often, these are patients with advanced non-small-cell lung carcinoma (NSCLC), with a histological subtype other than squamous-cell carcinoma (adenocarcinoma, large-cell carcinoma or mixed carcinoma with a predominance of these subtypes) or with a not-otherwise specified (NOS) histological subtype, in whom the presence of specific, somatic molecular alteration within cancer cells was confirmed. These include activating mutations in exons 18–21 of the *EGFR* (epidermal growth factor receptor) gene that are detected in approx. 40% Asian and 10% Caucasian (of which 25% in the American population, 15% in the European population) patients [1,2,3]. Over 188 different *EGFR* mutations have been identified, of which the 2 most common types are: small deletions within exon 19 (45–50%) and the p.(Leu858Arg) point mutation in exon 21 (40–45%), i.e., the so-called classic *EGFR* mutations [4]. The presence of any of these molecular alterations is a biomarker predicting the effectiveness of therapy with drugs from the group of EGFR tyrosine kinase inhibitors (EGFR-TKIs; erlotinib, gefitinib, afatinib, dacomitinib, osimertinib) [5]. Importantly, recent marketing authorization of a new drug for NSCLC treatment, amivantamab, targeting EGFR with exon 20 insertions (4–10% *EGFR*-positive cases) extends the list of molecular alterations that require accurate, sensitive and comprehensive analysis during the diagnostic process [6].

Targeted therapies in NSCLC patients with the above-mentioned genetic alterations are more effective than conventional chemotherapy, are associated with the improvement of the patients’ quality of life, and also show a favorable toxicity profile [7,8]. Thus, apart from radiological imaging and pathological examination, molecular diagnostics is the key phase of qualifying patients for targeted therapies that provides the necessary information about the status of molecular alterations within the tested genes of interest. The attending physician may consider starting targeted treatment only on the basis of a positive molecular test result.

Molecular diagnostics of lung cancer exploits a number of laboratory techniques enabling the effective analysis of clinically important genetic changes, in particular somatic mutations and gene rearrangements. Tumor tissue remains the main source of neoplastic genetic material (DNA, RNA) for testing. In recent decades, techniques using DNA amplification by real-time PCR (qPCR), and direct DNA sequencing by the Sanger method have been widely adopted for somatic mutation detection [9]. Nowadays, these techniques are being replaced by next-generation sequencing (NGS) characterized by a higher throughput, allowing for the sequencing of many regions within the genome at the same time, in a large number of samples (e.g., from different patients) for various genetic changes (point mutations, insertions, deletions, copy-number variation) within a single run [10]. Moreover, the detection sensitivity of the most popular NGS systems allows the detection at the level of 5% of the mutated allele in the background of the wild-type allele in DNA. Special protocols (e.g., for liquid biopsy) enable even more sensitive analysis, often below 1%. In addition, NGS is both a qualitative (reading DNA sequence, mutation detection) and quantitative method, as it provides the exact number of DNA reads supporting the presence of a given alteration.

In a routine diagnostic practice, focused, targeted-NGS panels detecting mutations within several appointed genes of clinical importance seem to show the highest robustness [11]. The adequacy of using broad molecular profiling, so called comprehensive genomic profiling (CGP), on a routine basis in NSCLC diagnosis is now under debate in terms of health care funding models, health technology assessment processes, reporting of complex genetic data to physicians and patients, and appropriate regulatory standards to determine the quality of genomic profiling tests [12,13]. The implementation of NGS into molecular diagnostics of lung cancer, however, should be preceded by the optimization and validation of the diagnostic procedures [14].

In this study, we evaluated the diagnostic usefulness of the focused targeted NGS panel for detection of actionable *EGFR* variants in a number of clinical tissue samples from advanced NSCLC patients previously analyzed using the In Vitro Diagnostic (IVD)-certified qPCR test with respect to the NSCLC cell lines and the biosynthetic DNA reference material. Additionally, point mutations in specific genomic regions of other genes associated with solid tumors, e.g., *BRAF*, *KRAS*, *PIK3CA*, and *TP53* (250 amplicons), were also analyzed.

## 2. Results

### 2.1. Overall Performance of qPCR and NGS Assays

#### 2.1.1. Cobas EGFR Mutation Test v2

The cobas EGFR mutation test v2 yielded valid results in all clinical samples. A (positive) mutant control and negative control were included in each run to confirm the validity of the run. Once the thermal run was complete, the software processed the raw data using dedicated analysis algorithms, and accepted the controls as valid based on cycle threshold (Ct) and relative Ct values for each sample and each channel.

#### 2.1.2. TruSight Tumor 15 Assay

NGS provided valid raw genomic data for all samples analyzed. All NGS reactions were conducted with high-quality parameters: QC30 range of 88.59–90.70% (quality norm according to the manufacturer—QC > 85%), cluster density spreading 1284 k/mm^2^–1544 k/mm^2^ (optimum according to the manufacturer 1200–1400 k/mm^2^). The median (min–max) total read depth (coverage) was 6649× (202–219,705) and the median (min–max) read depth of alleles that differed from the reference read (alt read depth) was 1667× (52–28,229) for all detected variants. For the *EGFR* variants, the median (min–max) total read depth was 6978× (387–219,705) and the median (min–max) read depth of alleles that differed from the reference read (alt read depth) was 2302× (52–28,230). In the DNA reference material, NGS assay demonstrated satisfactory inter-assay variability of the *EGFR* variant read frequency values between the runs. Median (min–max) coefficient of variations (CV%) were 1.44% (0.32–3.98%) in the biological DNA reference and 6.87% (0.43–13.41%) in the biosynthetic DNA reference material (Tables S1 and S2).

### 2.2. The NGS Detection Limit in DNA Reference Material

In DNA reference samples, all *EGFR* variants were correctly identified (Table 1). In the biosynthetic DNA reference material, NGS was capable of detecting variants down to the 3.3% allele frequency. The actual allelic frequencies of detected variants (p.(Glu746_Ala750del); p.(Asp770_Asn771insGly); p.(Thr790Met) matched the predicted values of 3.5%, 3.3%, and 3.3% well, except for the p.(Leu858Arg) variant due to diminished total read depth (468 reads). In the biological DNA reference material, actual allelic frequencies of detected *EGFR* variants (p.(Glu746_Ala750del); p.(Thr790Met); p.(Leu858Arg) corresponded well with predicted values of 10%, 5%, 2.5%, 1%. Analysis of reference DNA samples demonstrated sufficient sensitivity and specificity of TST15 assay below the detection limit of 5% allele frequency declared by the manufacturer.

### 2.3. The Detection of EGFR Variants in Clinical Samples by qPCR and NGS Assays

**Figure 1:**
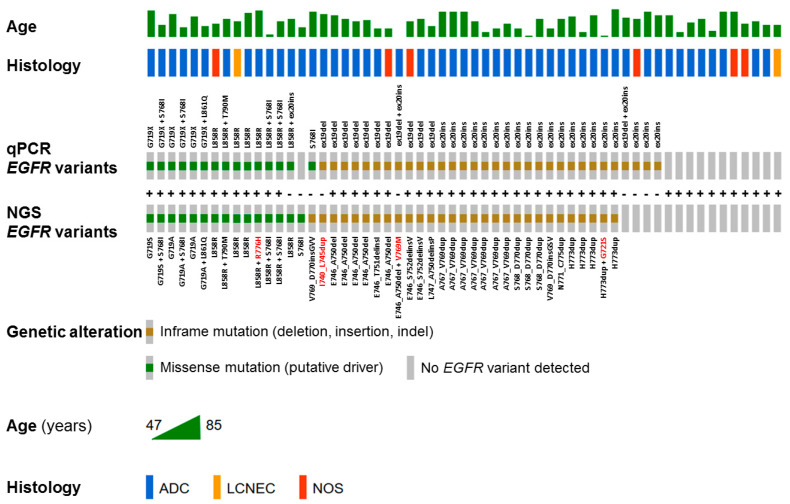
The OncoPrint showing the distribution of *EGFR* variants detected by qPCR cobas test and NGS TST15 assay in 59 NSCLC samples. The type of mutations are labeled in the color legend, particular *EGFR* variants in rows, and tumor samples in columns. The concordance/discordance between qPCR and NGS results were marked as “+” and “−”, respectively. The variants undetectable by qPCR test are highlighted in red color.

In total, 56 *EGFR* variants were reported by qPCR assay in comparison to 50 variants identified in the NGS analysis (Table S3). Both assays showed concordance in detection of p.(Gly719x) (*n* = 6), p.(Thr790Met) (*n* = 1), p.(Leu858Arg) (*n* = 8), and p.(Leu861Gln) (*n* = 1) variants. Major discrepancies concerned exon 19 deletions (*n* = 12 vs. 11), exon 20 insertions (*n* = 23 vs. 18), and p.(Ser768Ile) variants (*n* = 5 vs. 5 in different samples) identified by qPCR and NGS, respectively (Figure 1). A total of 9 out of 59 (15%) clinical samples (formalin-fixed, paraffin-embedded tissues, FFPET) showed discordant results for one or more *EGFR* variants in both assays (Table 2). In sample #7, the exon 19 deletion reported by cobas test was then identified as the rare exon 19 insertion in the NGS analysis. In samples #14, #18, #24, #39, #40, and #48, there were exon 20 insertions reported by cobas test which were not confirmed by NGS assay. Two samples, #19 and #32, showed a discordant status of p.(Ser768Ile) variant in both assays. Generally, the concordance rate between the qPCR test and NGS assay in *EGFR* variant detection and identification was 76.14% (Cohen’s Kappa = 0.5933). It is important to note that the coverage in *EGFR*-negative samples evaluated by NGS assay (including two samples where qPCR and NGS showed discrepancy for exon 20 insertion) was above the 500× threshold for all the amplicons, except a few suboptimal values for the 1EGFRxxE21TF035SR035.1 amplicon covering p.(Leu858Arg) and p.(Leu861Gln) variants (Table S4).

**Figure 2:**
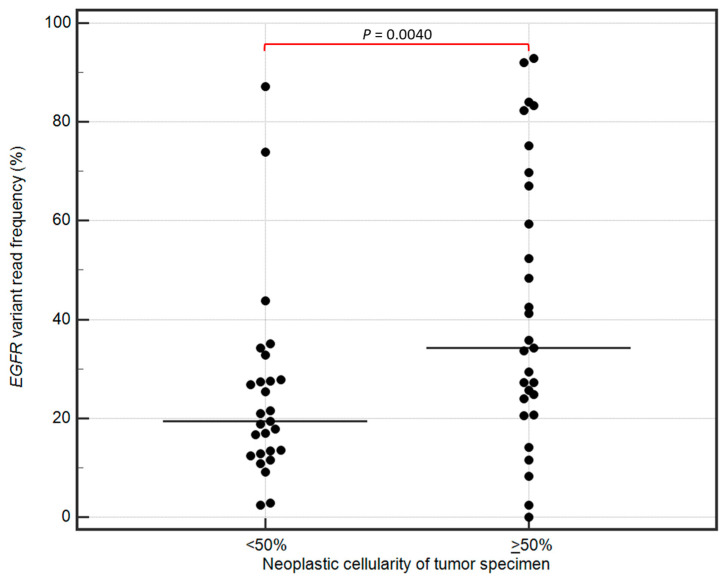
The association between the NGS read numbers and neoplastic cellularity of tumor specimens evaluated using nonparametric Mann–Whitney U test. The tumor specimens with neoplastic cellularity ≥ 50% showed significantly higher *EGFR* variant read frequencies (median 35.00; min–max: 2.46–92.82) than the specimens with cellularity < 50% (19.39; 2.44–87.16; *p* = 0.0040).

A Shapiro–Wilk analysis confirmed that the variables deviated significantly from a normal distribution (*p* < 0.05). Therefore, the association between the NGS read numbers and neoplastic cellularity of tumor tissue specimens were further evaluated using nonparametric Mann–Whitney U test. The tumor specimens with neoplastic cellularity ≥50% showed significantly higher *EGFR* variant read frequencies (median 35.00; min–max: 2.46–92.82) than the specimens with cellularity <50% (19.39; 2.44–87.16; *p* = 0.0040; Figure 2). Also, in Spearman’s correlation analysis, the neoplastic cellularity of tumor specimens was positively correlated with the *EGFR* variant read frequency (Spearman’s rank correlation coefficient = 0.40, *p* = 0.0046). There was no significant association between the neoplastic cellularity and the total read numbers in tumor specimens evaluated by NGS (*p* < 0.05).

### 2.4. Other Gene Variants Detected in Clinical Samples by NGS

Among the 59 clinical samples tested by NGS, *TP53* was the most frequently mutated gene in NSCLC patients, regardless their *EGFR* mutation status. A total of 27 out of 59 (46%) samples presented pathogenic/likely pathogenic *TP53* variant of which 21 had *EGFR* co-mutation (Table S3). Among 15 patients with *EGFR*-negative tumors, 4 patients had pathogenic *KRAS* variants (including two cases with p.(Gly12Cys) variant related to drug response), 2 patients had *BRAF* p.(Val600Glu) variant related to drug response, and 3 patients had pathogenic/likely pathogenic *PIK3CA* variants. No *KRAS* nor *BRAF* variants were detected in *EGFR*-positive tumor samples.

## 3. Discussion

The gradually increasing number of molecular therapeutic targets in NSCLC, on the one hand, creates new treatment opportunities for subsequent groups of patients presenting a specific type of genetic alteration. On the other hand, it constitutes a challenge for molecular diagnostics requiring the development and implementation of more and more effective analytical methods adapted to clinical realities in terms of the number and type of genetic alterations identified at the same time, as well as the cost-, time-, and labor-effectiveness of work. In recent decades, single-gene qPCR-based tests have been commonly used in laboratory diagnosis of somatic alterations within the *EGFR* gene in NSCLC patients because of their overall cost-effectiveness and robust results.

The cobas EGFR mutation test v2, a qPCR test, can identify 42 *EGFR* mutations in exons 18–21 (including the TKI-resistance mutation p.(Thr790Met); Tables S5 and S6). This test uses selective, allele-specific oligonucleotide primers and probes to detect each mutation; the probes are labeled with a fluorescent dye and fluorescence quencher molecule [15]. The cobas system contains sample preparation kits for manual DNA extraction from tissue or plasma (cobas DNA or cfDNA sample preparation kits, respectively) and uses qPCR to detect *EGFR* mutations.

In the manufacturer’s validation study, several FFPET specimen DNA extracts for the exon 19 deletions, p.(Leu861Gln), p.(Leu858Arg), p.(Thr790Met), p.(Gly719Ala), p.(Gly719Cys), p.(Gly719Ser), p.(Ser768Ile) variants, and exon 20 insertions were blended with *EGFR* wild-type FFPET specimen extracts to achieve blends with samples targeting 10, 5.0, 2.5 and 1.25% mutation level as determined by next-generation sequencing method [16]. The limit of detection of each sample was determined by the lowest amount of DNA that gave an *EGFR* “Mutation Detected” rate of at least 95% for the targeted mutation. This study demonstrates that the cobas test can detect mutations in *EGFR* exons 18, 19, 20, and 21 with at least 5% mutation level using the standard input of 50 ng per reaction well. The cobas EGFR Mutation Test (v1) was FDA-approved on May 14, 2013 for the qualitative detection of exon 19 deletions and exon 21 (p.(Leu858Arg)) substitution mutations of the *EGFR* gene in DNA derived from FFPE human NSCLC tumor tissue [17]. In 2015 the FDA approved cobas EGFR Mutation Test v2, adding, e.g., p.(Thr790Met) mutation to clinically important variants, identified up to now by above-mentioned original test [18]. The ‘gatekeeper’ p.(Thr790Met) mutation in *EGFR* exon 20 is the most common resistance mechanism to first- and second-generation EGFR-TKIs (erlotinib, gefitinib, afatinib, dacomitinib) that sterically hinders the binding of a small-molecule drug to the ATP-binding site of EGFR [19]. Currently, this test detects *EGFR* mutations in NSCLC patients whose tumors harbor the exon 18 (p.(Gly719Ala/Cys/Ser)) substitutions, exon 19 deletions, exon 20 insertions and substitutions (p.(Thr790Met), p.(Ser768Ile)) and exon 21 substitutions (p.(Leu858Arg), p.(Leu861Gln)), but not any other *EGFR* mutations (Tables S5 and S6) [20]. On June 1, 2016, FDA approved cobas EGFR Mutation Test v2 using plasma specimens as a companion diagnostic test for the detection of exon 19 deletions or exon 21 substitution mutations in the *EGFR* gene [21]. Several studies have demonstrated the cobas EGFR mutation test v2 to be superior over other qPCR-based tests in molecular analysis of *EGFR* mutations in tumor tissue [22,23,24] and liquid biopsy [25,26,27,28].

Nowadays, the molecular diagnostic process based solely on single-gene qPCR (for activating mutation detection) and FISH (for gene rearrangement detection) tests becomes less and less economically and clinically justified. Targeted therapies registered so far in NSCLC by the FDA and/or EMA target nine different oncoproteins, encoded by the genes *ALK, BRAF, EGFR, ERBB2, KRAS, MET, NTRK1–3, RET, ROS1* [29]. A total number of several hundred druggable molecular alterations are present in these genes. Furthermore, the very limited quantity of the neoplastic material available for molecular testing in advanced NSCLC is another important issue that favors testing multiple gene alterations simultaneously by NGS within the same tumor tissue sample over performing series of single-gene tests in different samples. In the report summarizing the state of molecular diagnostics of lung cancer in the years 2015–2021 in the United States, based on clinical data of 17,513 cases of advanced/metastatic non-squamous NSCLC, who underwent a total of 83,064 genetic tests, it was indicated that the percentage of patients diagnosed using the NGS method increased from 28.3% in 2015 to 68.1% in 2020 [30]. These data demonstrates well the actual global trend of molecular diagnostics towards high-throughput techniques offering simultaneous analysis of many alterations in a number of clinically important genes.

Following this trend, we demonstrate in this paper the advantages of using focused, targeted NGS panel for detection of actionable *EGFR* variants over the single-gene qPCR test as an example of potential benefits coming from switch to high-throughput method of molecular diagnostics. In our study, targeted NGS assay and the single-gene qPCR test showed 76.14% concordance rate (Cohen’s Kappa = 0.60) in detection of actionable *EGFR* variants in clinical tumor tissue specimens from 59 advanced NSCLC patients. The cobas EGFR mutation test v2 reported 56 *EGFR* variants in comparison to 50 variants identified in the NGS analysis. Major discrepancies concerned the numbers of exon 19 deletions, exon 20 insertions, and p.(Ser768Ile) variants identified by qPCR and NGS. The detailed analysis of nine out of 59 (15%) clinical samples (FFPET) showing discordant results for one or more *EGFR* variants in both assays revealed the higher tendency of the qPCR test to return false-positive results for exon 19 deletions and exon 20 insertions (Table 2). In three cases, the repeated cobas testing of a new FFPET section returned no variant in exons 19 and 20. In five cases, however, no extra tumor tissue was available to repeat qPCR testing as recommended by the manufacturer. A scarcity of tumor tissue available for molecular testing is a common issue in diagnostics of advanced NSCLC [31]. The identification of *BRAF* p.(Val600Glu) and *KRAS* p.(Gly12Cys) variants in two of those samples by NGS provided indirect evidence that the qPCR result could have been wrong due to general mutual exclusivity of class 1 *BRAF* mutations, *KRAS* codon 12 variants and activating *EGFR* mutations in NSCLC [32,33]. The clinical consequences of such false-positive results would lead, in the worst scenario, to administration of inadequate treatment in seven out of 9 discrepant cases. Similarly, the false-positive detection of exon 20 insertions by cobas EGFR mutation test v2 were observed with varying frequencies in other studies [34,35,36]. Interestingly, in one study, all false-positive results for exon 20 insertions were found in patients with squamous-cell carcinoma that even better visualize the problem of qPCR specificity since *EGFR* mutations are rare in well-characterized, fully excised surgical specimens of squamous-cell lung carcinoma lacking any adenocarcinoma component (reported frequency of less than 5%) [36,37]. The discordant p.(Ser768Ile) results between cobas EGFR mutation test v2 and other diagnostic platforms were also reported elsewhere showing the tendency of the ‘cobas’ diagnostic qPCR system to return this variant either as false-positive [38,39,40] or false-negative [23,41] due to possible intrinsic (mismatch in probe binding site) and extrinsic (presence of PCR inhibitors, low DNA integrity) limitations.

In our laboratory setting, targeted NGS assay demonstrated sufficient analytical sensitivity below the detection limit of 5% allele frequency declared by manufacturer. In DNA reference samples, all *EGFR* variants were correctly identified and the actual allelic frequencies of detected variants matched the predicted values well. In the clinical samples, NGS was capable of detecting *EGFR* variants at the level of approx. 2.5% allele frequency. The median (min–max) total read depth for the *EGFR* variants was 6978× (387–219,705). However, the detection of variants at low allelic frequencies (<2–3%) may be affected by a high risk of a false-positive result, regardless of the coverage depth, since a conventional intrinsic NGS error rate for MiSeq platform is about 0.5% [42]. Using the simple theoretical coverage limit calculator by OLGEN, we calculated the recommended minimum coverage to be 1131× for variants detected at 2.5% allelic frequency while 356× for variants detected at 5% allelic frequency, adopting the 0.5% sequencing error rate and 0.1% probability of false-positive result [43]. Thus, in theory, we could report *EGFR* variants at 2.5% allelic frequency when taking into account the total read depth achieved in this study. In practice, however, the sequencing error rate, apart from errors produced by sequencing itself, includes other errors introduced during DNA processing and library preparation, particularly during amplification steps, which further increase error rates [44].

There is currently no consensus on the minimum required coverage for somatic variant detection in a clinical setting using targeted NGS. It is recommended that each laboratory has to set and validate its own parameters in order to meet sufficient quality in somatic variant detection by NGS [45,46,47]. In molecular pathology of solid tumors, 300 to 500 sequence reads per target are usually sufficient to cover almost all diagnostic alterations if derived from sufficient template molecules [45]. In our study, we showed that the *EGFR* variant read frequency was significantly lower in the specimens with neoplastic cellularity < 50% though the total read numbers per target remained similar to values presented by samples with ≥50% cellularity. This happens because malignant lung tumors are highly heterogeneous and the actual limit of detection of NGS depends on the percentage of available tumor cells. According to the literature, in approximately 30% of patients diagnosed with NSCLC, tumor cellularity is <40% due to the small amount of tissue derived from biopsy [48]. Therefore, setting the limit of detection at 5% allelic frequency and the minimum coverage at 500× provides the safety margin that reduce the risk of erroneous results in samples presenting high neoplastic cellularity. To detect and not miss clinically important gene alterations in samples with low tumor cellularity, it may be necessary to increase the coverage [45]. Importantly, a special caution should be taken in interpreting negative NGS results. For true-negative results, it is essential to ensure that the coverage in *EGFR*-negative samples is above the 500× (or higher) threshold for all the amplicons, as it has been demonstrated in our study.

Major advantages of targeted NGS over multiplex qPCR tests are the exact identification of a variant sequence and the measurement of the proportion of variant alleles within a genomic locus reported as the variant allele frequency (VAF) or variant read frequency. Recent studies using NGS shed more light on the actual impact of *EGFR* exon 19 deletion subtypes and VAF on clinical outcomes in EGFR-TKI-treated advanced non-small-cell lung cancer. In the meta-analysis including a total of eleven retrospective studies and one prospective study involving 1630, mostly Asian NSCLC patients with *EGFR* exon 19 deletions, deletions starting from E746 were significantly associated with longer OS than those with deletions starting from L747 (HR, 0.79; 95% CI: 0.65 to 0.96, *p* = 0.019), and relatively but not significantly longer PFS (HR, 0.86; 95% CI: 0.69 to 1.06, *p* = 0.160) [49]. Patients with E746_A750del, the most common exon 19 deletion subtype (55% of all deletions in our study) had a significantly higher frequency of acquired p.(Thr790Met) mutation when treated with first- or second-generation EGFR-TKIs compared to those with other exon 19 deletions subtypes (RR, 0.76; 95% CI: 0.64–0.89, *p* = 0.001). No differences in PFS between the E746_A750del group and the uncommon group, or between the 15-nucleotide deletion group and other patients were observed. Moreover, deletions occurring in the C-helix part of the *EGFR* exon 19 that constitute about 2.5% of all exon 19 deletions were associated with the best response as partial response rate (72.7%), and the progression-free survival (PFS) of 12.0 months in a cohort of 1138 advanced NSCLC patients treated with EGFR-TKIs [50]. However, the C-helix exon 19 deletions could be undetected by routine testing based on the single-gene qPCR assays, which often does not cover the whole spectrum of exon 19; only one such C-helix deletion (p.S752_I759del) is covered by cobas EGFR mutation test v2 (no cases identified in our study).

Currently, the research is underway to assess the predictive value of VAF in NSCLC patients presenting activating *EGFR* variants at different VAF levels in the NGS analysis. In detail, VAF refers to the proportion of sequencing reads that support a specific variant allele relative to the total number of reads within a genomic locus [51]. The available results, though limited by low numbers of patients, indicate a clear PFS improvement in NSCLC patients treated with EGFR-TKIs who present *EGFR* mutations at a high level of VAF. The PFS among 31 patients treated with front-line TKIs (gefitinib, erlotinib, afatinib, dacomitinib, osimertinib) with a mutant allelic frequency ≤ 9% was 92 days, compared to 284 days for those with a frequency greater than 9% (*p* = 0.0027), suggesting a predictive role of this variable [52]. Likewise, in 89 NSCLC patients receiving either gefitinib or erlotinib as first- or second-line systemic therapy, a statistically significant positive linear correlation was found between adjusted (normalized to the proportion of neoplastic cells in each specimen) VAF of *EGFR* variant in tumoral tissue (aVAF) and PFS (r = 0.319; *p* = 0.002) [53]. High (≥70%) *EGFR*-aVAF was an independent predictor of longer PFS [vs. low (<70%) *EGFR*-aVAF; median PFSs were 52 vs. 26 weeks, respectively; *p* < 0.001]. Additionally, patients with high *EGFR*-aVAF also had significantly improved OS than those with low *EGFR*-aVAF (*p* = 0.011). In other study, low VAF < 0.25 was significantly associated with worse PFS of osimertinib (8.4 months [95% confidence interval (CI): 0.4 months—not reached]) when compared to patients presenting *EGFR* variants at high VAF ≥ 0.25 (median not reached; (*p* = 0.032, hazard ratio 4.9 [95% CI: 0.86–28.2]) [54].

In our study cohort, *TP53* was the most frequently mutated gene and twenty seven out of 59 (46%) patients presented pathogenic/likely pathogenic *TP53* variant of which 21 had *EGFR* co-mutation. To date, many studies investigating the association between *TP53* co-mutations and patient responses to single-agent treatment with EGFR-TKIs demonstrated the negative impact of those alterations on survival outcomes in advanced NSCLC [55]. For example, Hou et al. showed significantly shorter median PFS (6.5 vs. 14 months, *p* = 0.025) and median overall survival (OS, 28 vs. 52 months, *p* = 0.023) in advanced NSCLC patients with the *TP53* co-mutations treated with the first-generation TKIs (gefitinib, erlotinib, and icotinib) with respect to the patients with the wild-type *TP53* [56]. *TP53* mutations demonstrated a negative impact on PFS and OS in a group of patients carrying a sensitizing *EGFR* mutation and a p.(Thr790Met) resistance mutation treated with osimertinib in 2nd line [57]. PFS for *TP53*-mutant patients were 9 months vs. 14 months for patients with *TP53* wild-type (*p* < 0.008). OS for *TP53*-mutant patients was 16 months vs. 24 months patients with *TP53* wild-type (*p* < 0.025).

This study has several limitations. The total number of 59 analyzed NSCLC cases may seem to be small, but the study cohort includes 18 patients with *EGFR* exon 20 insertions, and major discrepancies between NGS and qPCR results were observed in this subgroup. Given that seven out of 24 (29%) reported results for exon 20 insertions were discordant between NGS and qPCR, the clinical importance of this diagnostic issue can be easily extrapolated on larger number of patients. We did not evaluate the response and survival endpoints (ORR, PFS, OS) in NSCLC patients presenting different types of *EGFR* mutations as such data have already been reported elsewhere [58,59], and here we focused on diagnostic issues only.

## 4. Materials and Methods

### 4.1. Patients

In total, 59 NSCLC patients (median age: 69 years; range: 47–85 years) were included into this study. The histologic subtypes of NSCLC were determined according to the World Health Organization classification [60,61]. Of the included patients, 51 (86.5%) had adenocarcinoma (ADC), 2 (3.5%) had large-cell neuroendocrine carcinoma of the lung (LCNEC), and 6 (10%) had cancers not otherwise specified (NOS). All NSCLC patients had advanced metastatic disease (stages IIIB–IV) according to the tumor node metastasis (TNM) international staging system [62]. None of the patients received treatment prior to mutation testing. The molecular testing for *EGFR* mutations was performed in patients with advanced non-squamous-cell carcinoma according to diagnostic and therapeutic strategy for NSCLC patients in Poland and current guidelines of the European Society for Medical Oncology (ESMO) [63]. Six (10%) patients had *EGFR* p.(Gly719Ala/Cys/Ser) variant, 10 (17%) patients had exon 19 deletions (ex19del), 18 (30.5%) patients had exon 20 insertions (ex20ins), 8 (13.5%) patients had exon 21 p.(Leu858Arg) substitutions, and 7 (12%) patients had other *EGFR* mutations (p.(Ser768Ile), p.(Leu861Gln)). Fifteen (25.5%) patients presented no *EGFR* mutation in their tumors. One patient (1.5%) with p.(Leu858Arg) activating *EGFR* mutation presented p.(Thr790Met) co-mutation at diagnosis. All patients were Caucasian. The study was conducted after approval from the local ethics committee at the Institute of Tuberculosis and Lung Diseases in Warsaw, Poland (KB-56/2022). The demographic and clinical characteristics of the patients are summarized in Table 3.

### 4.2. Clinical Tumor Specimens

The tumor tissue specimens were acquired from the NSCLC patients during surgery or biopsy, and standard clinical procedures were followed. In total, 51 (86.5%) FFPE tumor tissue samples and 8 (13.5%) cytological smears were analyzed for molecular alterations. In each sample chosen for qPCR and/or NGS, an experienced pathologist estimated by eye under a light microscope the proportion of tumor cells relative to all nucleated cells. The median (range) tumor cell content was 50% (10–100%) in FFPE tissues and 85% (10–100%) in cytological smears.

### 4.3. Lung Cancer Cell Lines and In Vitro Culture Conditions

Five human cell lines: H2347 (CRL-5942), HCC4006 (CRL-2871), H1975 (CRL-5908), H1650 (CRL-5883), PC-9 (90071810) were purchased from American Type Culture Collection (ATCC; LGC Standards, Kielpin, Poland) and European Collection of Authenticated Cell Cultures (ECACC; Public Health England, Salisbury, UK). All the cell lines have epithelial morphology and represent the adenocarcinoma subtype of non-small-cell lung cancer. They were established from primary (H2347, PC-9, H1975) or metastatic tumor tissue (HCC4006, H1650) explanted from the NSCLC patients.

The lung cancer cell lines were grown in polystyrene flasks with filter cap (Sarstedt AG & Co. KG, Nümbrecht, Germany; cat. no. 83.3910.002) as adherent monolayers in RPMI-1640 (ATCC; LGC Standards, Kielpin, Poland; cat. no. 30-2001) medium supplemented with 10% fetal bovine serum (cat. no. 04-121-1B) and 1% antibiotics (penicillin, streptomycin and amphotericin B; cat. no. 03-033-1; Biological Industries, Kibbutz Beit-Haemek, Israel ), in temperature of 37 °C, at 5% CO_2_ saturation. Each cell line was cultured in the two biological replicates. The characteristics of the NSCLC cell lines has been presented in Table S7.

### 4.4. DNA Extraction

The cultured cells were harvested at >90% confluency by trypsin-EDTA treatment (Biological Industries, Kibbutz Beit-Haemek, Israel; cat. no. 03-079-1B) to 15 mL falcon tube and washed twice in a sterile Dulbecco’s Phosphate Buffered Saline (PBS) buffer without Ca^2+^ and Mg^2+^ ions (Biological Industries, Kibbutz Beit-Haemek, Israel; cat. no. 02-023-1A). DNA was extracted from approximately 1 × 10^6^ cells using the QIAamp DNA Mini Kit (Qiagen, Hilden, Germany; cat. no. 51304) according to manufacturer’s instruction.

FFPET blocks were cut into 5 μm-thick sections and collected in sterile Eppendorf tubes. DNA was extracted from the FFPE tumor tissue sections using the spin column-based cobas DNA sample preparation kit (Roche Diagnostics GmbH, Mannheim, Germany; cat. no. 05985536190), in accordance with the manufacturer’s instruction.

Cytological specimens were prepared by manual scraping tumor cells from smear slides into sterile Eppendorf tubes. DNA was extracted from collected cell using the QIAamp DNA Micro Kit (Qiagen, Hilden, Germany) according to the manufacturer’s instruction.

Buffy coats were isolated from peripheral blood of healthy volunteers by centrifugation for 10 min at 1600× *g*. DNA was extracted from buffy coats using the QIAamp DNA Micro Kit (Qiagen, Hilden, Germany; cat. no. 56304) according to the manufacturer’s instruction.

The concentration and purity of DNA eluates were measured by spectrophotometry (for qPCR) using the NanoVue Plus spectrophotometer (GE Healthcare UK Limited, United Kingdom) and fluorometry (for NGS) using the QuantiFluor ONE dsDNA System reagent (cat. no. E4870) and Quantus fluorometer (Promega Corporation, Madison, WI, USA), in accordance with the manufacturers’ protocols and laboratory guidelines [47,64].

### 4.5. Biosynthetic DNA Reference Material

The Seraseq Tri-Level Tumor Mutation DNA Mix v2 (material number: 0710-0097, batch number: 10621387, LGC SERACARE, Milford, MA, USA) reference DNA material was used in NGS analysis to test the sensitivity and specificity of the targeted assay (Figure S1). This product is a multiplexed mixture of actionable biosynthetic DNA targets precisely blended with a single, well-characterized human genomic DNA as background ‘wild-type’ material and presenting a range of allele frequencies distributed at 4%, 7% or 10%, including *EGFR*, *BRAF*, *KRAS*, *ERBB2*, and *TP53* variants (Table S1). 

### 4.6. Biological DNA Reference Material

Control samples were prepared by mixing DNA from NSCLC cell lines with known *EGFR* mutation status and allelic frequency: PC-9 (ECACC, 90071810; *EGFR* ex19del p.(Glu746_Ala750del)) and H1975 (ATCC, CRL-5908; *EGFR* p.(Leu858Arg) + p.(Thr790Met)). DNA extracted from cell lines was diluted with DNA isolated from buffy coat of healthy volunteers (wild-type *EGFR*) to obtain approximate allelic frequencies of 10%, 5%, 2.5% and 1% for the mutant allele. Additionally, DNA extracted from all five NSCLC cell lines was analyzed by targeted NGS.

### 4.7. EGFR Mutation Analysis in Clinical Samples by Multiplex qPCR

All clinical samples were tested for *EGFR* mutations using the cobas EGFR mutation test v2 (cat. no. 07248563190) and the cobas z 480 Analyzer (Roche Diagnostics GmbH, Mannheim, Germany), in accordance with the manufacturer’s instructions. This test can identify 42 different mutations that may be present in exons 18, 19, 20 and 21 of the *EGFR* gene, including the p.(Thr790Met) resistance mutation at ≥ 5% mutation level in FFPET specimen DNA blends, according to the product insert provided by the manufacturer.

### 4.8. NGS Analysis of Gene Variants in Cell Lines and Clinical Samples

The comprehensive assessment of single-nucleotide variants (SNVs) and insertions/deletions (INDELs) in 15 genes that are commonly mutated in solid tumors was performed by targeted next-generation sequencing (the complete gene list in Table S8). Briefly, the DNA libraries were constructed using the TruSight Tumor 15 assay (TST15, Illumina, Inc., San Diego, CA, USA, cat. no. OP-101-1001). The concentration of each NGS library was measured by fluorimetry using the QuantiFluor ONE dsDNA System reagent (cat. no. E4870) and the Quantus fluorometer (Promega Corporation, Madison, WI, USA). The quality check of the NGS libraries was performed by running an aliquot of each normalized library on a 2% agarose gel electrophoresis using 50 bp DNA ladder. The libraries meeting the qualitative and quantitative requirements were sequenced on the MiSeq instrument (Illumina) using the high-output MiSeq Reagent Kit v3 (cat. no. MS-102-3003, Illumina) at a 2 × 150 bp read length configuration and dual indexing. This technique allows for sensitive and accurate detection of somatic variants with a 5% allele frequency using 20 ng DNA input at minimum 500× coverage.

### 4.9. Bioinformatics and Computational Analysis of NGS Output Data

The processing of the next-generation sequencing output data, including such steps as demultiplexing, alignment and variant calling, was performed using the MiSeq Reporter v2.6 software (Illumina). The obtained gVCF files (*.genome.vcf) were further analyzed using the BaseSpace Variant Interpreter application (Illumina) for variant annotation and filtering, and the reference genome version hg19/GRCh37. BaseSpace Variant Interpreter comprises a broad range of annotation sources (dbSNP, COSMIC, ClinVar, 1000 Genomes Project, EVS, ExAC, PolyPhen, SIFT, and OMIM) and provides annotations at variant, transcript, and gene levels for comprehensive assessment of the biological impact of genetic variation including prediction of transcript consequence (to segregate synonymous from different types of nonsynonymous changes), functional impact (to indicate variants that are likely deleterious), conserved sequence (to denote sequence similarity if the variant occurs between species), disease association. Highly confident variants were filtered out from the falsely called ones according to variant quality (read depth ≥ 200 and variant allele frequency ≥ 0.02), variant’s position relative to the transcript (coding variants vs. intronic/UTR5/UTR3 variants), genetic variation (removing SNPs with identical calls in all the sample), functional impact (nonsynonymous vs. synonymous and nonsense mutations), and pathogenicity (likely-/pathogenic vs. likely-/benign and variants of uncertain significance, VUS), adopted from the ComPerMed Expert Panel recommendations for somatic variant classifications in solid tumors [65,66].

All detected variants were verified for their impact on the coding region (missense, nonsense, frameshift mutations) and clinical significance (pathogenic, likely pathogenic, benign, likely benign, uncertain) according to the ClinVar [67] and the VarSome databases [68]. The synonymous variants were filtered out and not included into the final analysis. The required sequence coverage depth for a given n-percent somatic mutation and a defined error rate of measurement was calculated using theoretical coverage limit calculator [43]. The variant annotation was performed according to the standardized Human Genome Variation Society (HGVS) nomenclature [69]. Variants are described in the main text according to the HGVS protein sequence name (HGVSp) or using HGVSp short form to improve clarity. The distribution of *EGFR* variants detected by qPCR and NGS in NSCLC samples was generated using the OncoPrinter tool available in cBioPortal v. 4.0.2 [70,71].

### 4.10. Statistical Analysis

The standard deviation (SD) and the coefficient of variation (CV) were calculated for the variant read frequency from the two independent NGS measurements (run #1 and run #2) to assess the precision (repeatability) of the method in the reference material. The *EGFR* mutation detection rates from qPCR and NGS were analyzed using 2 × 2 tables in the MedCalc Statistical Software version 20.109 (Ostend, Belgium) to determine the concordance rate and Cohen’s Kappa coefficient (κ) [72,73]. The normality of the data was assessed using the Shapiro–Wilk test, and the nonparametric Mann–Whitney U test was used to compare the means. Spearman’s rank correlation test was used to analyze the association between the variables. The *p*-values were two tailed, and *p* < 0.05 was considered statistically significant.

## 5. Conclusions

In our study, the targeted-NGS assay showed a number of superior features over the single-gene qPCR in detection and identification of druggable EGFR variants (exact identification of EGFR variants, calculation of allelic frequency, high analytical sensitivity), which might enhance the basic diagnostic report. The implementation of such NGS techniques into the molecular diagnosis of lung cancer brings obvious benefits for the patients and the clinicians, despite the seemingly high cost of a diagnostic tests using the NGS method (in economic, equipment, personal and time terms).

The technical and clinical issues related to performing molecular diagnostics of lung cancer based on NGS techniques are more and more frequently addressed in recommendations, opinions and guidelines of domestic and international scientific societies and expert teams published in recent years. It is worth mentioning, for example, the recommendations of specialists from Portugal [74], Italy [75], Spain [76], practical guidelines of The Japanese Society of Pathology [77], The Korean Cardiopulmonary Pathology Study Group [78], recommendations of the ESMO Precision Medicine Working Group [79], joint guidelines of the Association for Molecular Pathology and the College of American Pathologists [80], as well as the International Association for the Study of Lung Cancer and the Association for Molecular Pathology [81].

## Figures and Tables

**Table 1:** The summary of single-nucleotide variants identified in the biosynthetic DNA reference (Seraseq, LGC Seracare, Milford, MA, USA) and the biological DNA reference material (NSCLC cell lines) using TST15 panel and MiSeq platform (Illumina, Inc., San Diego, CA, USA) for targeted NGS.

Reference DNA Material					NGS TST15 Results		
Gene	HGVSc	HGVSp	COSMIC ID	Average Allele Frequency	Detection Status [Yes/No]	Total Read Depth *	Variant Read Frequency *
Biosynthetic DNA reference material (Seraseq)							
*EGFR*	c.2236_2250del	p.(Glu746_Ala750del)	COSM6225	3.5%	Yes	1283	3.50%
	c.2310_2311insGGT	p.(Asp770_Asn771insGly)	COSM12378	3.3%	Yes	5320	2.28%
	c.2369C > T	p.(Thr790Met)	COSM6240	3.3%	Yes	5325	2.26%
	c.2573T > G	p.(Leu858Arg)	COSM6224	7.3%	Yes	468	2.56%
Biological DNA reference material (NSCLC cell lines)							
*EGFR*	c.2236_2250del	p.(Glu746_Ala750del)	COSM6225	10%	Yes	1719	14.54%
				5%	Yes	1665	5.35%
				2.5%	Yes	1508	4.91%
				1%	Yes	1535	2.41%
*EGFR*	c.2369C > T	p.(Thr790Met)	COSM6240	10%	Yes	10,925	15.13%
				5%	Yes	9955	7.47%
				2.5%	Yes	8250	4.73%
				1%	Yes	9990	1.4%
*EGFR*	c.2573T > G	p.(Leu858Arg)	COSM6224	10%	Yes	440	16.82%
				5%	Yes	510	5.1%
				2.5%	Yes	568	2.46%
				1%	Yes	502	3.19%

* Mean value from run #1 and run #2.

**Table 2:** The summary of discrepant results from *EGFR* variant analysis using the ‘cobas’ diagnostic qPCR system (Roche Diagnostics GmbH, Mannheim, Germany), and TST15 panel and MiSeq platform (Illumina, Inc., San Diego, CA, USA) for targeted next-generation sequencing (NGS).

Sample No.	Cobas qPCR Result	NGS Result (HGVSp)	Explanation of Discrepancy	EGFR-TKI Administered
7	ex19del	p.(Ile740_Lys745dup)	Rare exon 19 insertion erroneously interpreted as deletion by cobas due to mismatch in probe binding site. Sensitizing *EGFR* mutation.	osimertinib
14	ex19del	p.(Glu746_Ala750del)	Concordant.	gefitinib
	ex20ins	n/d	False-positive result in cobas analysis. Variant *EGF*R c.2305G > A p.(Val769Met) detected in NGS analysis is possible cause of mismatch at position 2305 falsely interpreted as ex20ins by cobas. Repeated cobas analysis of new FFPET section returned no ex20ins.	
18	ex19del	n/d	Both results false-positive in cobas analysis. Repeated cobas analysis of new FFPET section returned no ex19del and no ex20ins.	No
	ex20ins	n/d		
19	n/d	p.(Ser768Ile)	Discordant results. No tumor tissue available to repeat test.	No
24	ex20ins	n/d	False-positive result in cobas analysis. No tumor tissue available to repeat cobas analysis. In NGS analysis *BRAF* p.(Val600Glu) present (mutually exclusive with *EGFR* mutations).	No
32	S768I	p.(Val769_Asp770insGlyValVal)	Discordant results. No tumor tissue available to repeat test. The proximity of ex20ins to the S768I may be a possible cause of mismatch at position 768.	No *
39	ex20ins	n/d	False-positive result in cobas analysis. No tumor tissue available to repeat test.	No
40	ex20ins	n/d	False-positive result in cobas analysis. No tumor tissue available to repeat test. *KRAS* p.(Gly12Cys) present and mutually exclusive with *EGFR* mutations.	No **
48	L858R	p.(Leu858Arg)	Concordant.	osimertinib
	ex20ins	n/d	False-positive result in cobas analysis. Repeated cobas analysis of new FFPET section returned no ex20ins.	

* At the time of diagnosis, amivantamab, a bispecific monoclonal antibody currently registered with indication for *EGFR* exon 20 insertion-positive non-small-cell lung cancer, was not yet FDA/EMA-approved. ** At the time of diagnosis, neither sotorasib nor adagrasib, small-molecule inhibitors currently registered with indication for *KRAS* p.(Gly12Cys)-positive non-small-cell lung cancer, was not yet FDA/EMA-approved.

**Table 3:** The clinicopathological characteristics of 59 NSCLC patients from whom tumor specimens were obtained for molecular analysis.

Characteristics		*n* (%)
NSCLC patients		59 (100%)
Median age (range), years	68 (47–85)	
Sex		
Female		37/59 (63%)
Male		22/59 (37%)
Histology (WHO)		
ADC		51/59 (86.5%)
LCNEC		2/59 (3.5%)
NOS		6/59 (10%)
Stage IIIb–IV (TNM)		59/59 (100%)
*EGFR* mutation status		
	No mutation detected	15/59 (25.5%)
	Exon 18 p.(Gly719Ala/Cys/Ser)	6/59 (10%)
	Exon 19 deletion	10/59 (17%)
	Exon 20 insertion	18/59 (30.5%)
	Exon 20 p.(Thr790Met)	1/59 (1.5%)
	Exon 21 p.(Leu858Arg)	8/59 (13.5%)
	Other	7/59 (12%)

## Data Availability

The detailed data presented in this study are available from the corresponding authors on reasonable request.

## Supplementary Materials

The following supporting information can be downloaded at: https://www.mdpi.com/article/10.3390/ijms25147908/s1.
