# Supplementary material for: The Advantage of Targeted Next-Generation Sequencing over qPCR in Testing for Druggable EGFR Variants in Non-Small-Cell Lung Cancer
Source: Int J Mol Sci. 2024 Jul 19;25(14):7908. doi: 10.3390/ijms25147908 (PMC11277480; doi:10.3390/ijms25147908)
Supplement: Supplementary file 1 [file ijms-25-07908-s001.zip › ijms-3103240-supplementary.pdf]

SUPPLEMENTARY MATERIAL

Table S1. The results of the biosynthetic DNA reference (Seraseq, LGC Seracare, Milford, MA, USA) sequencing using TST15 panel and MiSeq platform (Illumina, Inc., San Diego, CA, USA).

Biosynthetic DNA reference material (Seraseq)					NGS TST15 results						
Gene	HGVSc	HGVSp	COSMIC ID	Average dPCR allele frequency	Detection status [Yes/No]	Total Read Depth		Variant Read Frequency			
						Run #1	Run #2	Run #1	Run #2	SD	CV (%)
4% Tier											
BRAF	c.1799T>A	p.(Val600Glu)	COSM476	4.5%	Yes	12900	11842	2.55%	2.62%	0,04	1,35
EGFR	c.2310_2311insGGT	p.(Asp770_Asn771insGly)	COSM12378	3.3%	Yes	5138	5502	1.97%	2.58%	0,31	13,41
	c.2236_2250del	p.(Glu746_Ala750del)	COSM6225	3.5%	Yes	1204	1362	3.49%	3.52%	0,01	0,43
	c.2369C>T	p.(Thr790Met)	COSM6240	3.3%	Yes	5510	5141	2.54%	1.98%	0,28	12,39
FOXL2	c.402C>G	p.(Cys134Trp)	COSM33661	4.5%	Yes	4511	4143	7.10%	8.13%	0,52	6,76
PDGFRA	c.1694_1695insA	p.(Ser566GlnfsTer6)	COSM28053	3.1%	Yes	10435	13113	2.57%	2.36%	0,11	4,26
PIK3CA	c.3140A>G	p.(His1047Arg)	COSM775	4.0%	Yes	10894	9505	2.66%	2.41%	0,13	4,93
	c.3204_3205insA	p.(Ter1069MetfsTer4)	COSM12464	3.8%	Yes	10892	9513	2.66%	2.41%	0,13	4,93
RET	c.2753T>C	p.(Met918Thr)	COSM965	3.8%	Yes	17794	17462	2.16%	2.38%	0,11	4,85
TP53	c.267del	p.(Ser90ProfsTer33)	COSM18610	Not assayed	Yes	15338	14920	1.98%	2.14%	0,08	3,88
7% Tier											
EGFR	c.2573T>G	p.(Leu858Arg)	COSM6224	7.3%	Yes	468	n/d	2.56%	n/d		
KRAS	c.35G>A	p.(Gly12Asp)	COSM521	7.7%	Yes	4238	4341	5.00%	4.91%	0,04	0,91
NRAS	c.182A>G	p.(Gln61Arg)	COSM584	7.3%	Yes	7421	6349	5.07%	4.93%	0,07	1,40
TP53	c.524G>A	p.(Arg175His)	COSM10648	7.2%	Yes	13195	12321	4.99%	4.54%	0,23	4,72
TP53	c.723del	p.(Cys242AlafsTer5)	COSM6530	6.8%	Yes	15992	18755	4.39%	4.26%	0,06	1,50
TP53	c.743G>A	p.(Arg248Gln)	COSM10662	7.3%	Yes	15945	18724	4.51%	4.30%	0,11	2,38
TP53	c.818G>A	p.(Arg273His)	COSM10660	7.1%	Yes	4949	4303	3.44%	3.25%	0,10	2,84
10% Tier											
AKT1	c.49G>A	p.(Glu17Lys)	COSM33765	9.6%	Yes	34267	35390	10.98%	10.74%	0,12	1,10
ERBB2	c.2313_2324dup	p.(Tyr772_Ala775dup)	COSM682	8.1%	Yes	11678	9461	8.52%	8.77%	0,13	1,45
GNA11	c.626A>T	p.(Gln209Leu)	COSM52969	9.4%	Yes	11924	13152	9.77%	9.50%	0,14	1,40
GNAQ	c.626A>C	p.(Gln209Pro)	COSM28758	10.3%	Yes	4277	4612	12.11%	11.59%	0,26	2,19
KIT	c.2447A>T	p.(Asp816Val)	COSM1314	10.4%	Yes	6062	6379	11.33%	11.57%	0,12	1,05
PDGFRA	c.2525A>T	p.(Asp842Val)	COSM736	10.9%	Yes	12161	13504	10.32%	10.70%	0,19	1,81
PIK3CA	c.1633G>A	p.(Glu545Lys)	COSM763	9.7%	Yes	5585	6294	8.53%	8.59%	0,03	0,35

Abbreviations: *AKT1* – AKT Serine/Threonine Kinase 1; *BRAF* – B-Raf Proto-Oncogene, Serine/Threonine Kinase; *EGFR* – Epidermal Growth Factor Receptor; *ERBB2* – Erb-B2 Receptor Tyrosine Kinase; *FOXL2* – Forkhead Box L2; *GNA11* – G Protein Subunit Alpha 11; *GNAQ* – G Protein Subunit Alpha Q; *KIT* – KIT Proto-Oncogene, Receptor Tyrosine Kinase; *KRAS* – Protein V-Ki-ras2 Kirsten rat sarcoma viral oncogene homolog; *MET* - MET Proto-Oncogene, Receptor Tyrosine Kinase; *NRAS* – NRAS Proto-Oncogene, GTPase; *PDGFRA* – Platelet Derived Growth Factor Receptor Alpha; *PIK3CA* – Phosphatidylinositol-4,5-Bisphosphate 3-Kinase Catalytic Subunit Alpha; *RET* – Ret Proto-Oncogene; *TP53* – Tumor protein P53.

Table S2. The summary of single nucleotide variants identified in the biological DNA reference from NSCLC cell lines using TST15 panel and MiSeq platform (Illumina, Inc., San Diego, CA, USA) for targeted NGS.

Cell line	Gene	HGVSC	HGVSP	Exon	dbSNP	Variant type	Total Read Depth		Variant Read Frequency			
							Run #1	Run #2	Run #1	Run #2	SD	CV (%)
**HCC4006**	*EGFR*	c.2239_2248delTTAAGAGAAGinsC	p.(Leu747_Ala750delinsPro)	19/28	rs727504278	indel	3685	1382	77.26%	71.35%	2.95	3.98
	TP53	c.613T>C	p.(Tyr205His)	6/11	rs1057520008	missense	14895	14104	99.81%	99.89%	0.04	0.04
**PC-9**	*EGFR*	c.2235_2249delGGAATTAAGAGAAGC	p.(Glu746_Ala750del)	19/28	rs121913421	inframe deletion	3429	1318	82.12%	85.13%	1.51	1.80
	TP53	c.743G>A	p.(Arg248Gln)	7/11	rs11540652	missense	20361	23690	99.89%	99.92%	0.02	0.02
**H1975**	*EGFR*	c.2573T>G	p.(Leu858Arg)	21/28	rs121434568	missense	956	868	81.99%	82.52%	0.27	0.32
		c.2369C>T	p.(Thr790Met)	20/28	rs121434568	missense	11195	7853	78.79%	80.06%	0.63	0.80
	TP53	c.818G>A	p.(Arg273His)	8/11	rs28934576	missense	3511	3256	99.83%	99.82%	0.01	0.01
**H1650**	*EGFR*	c.2235_2249delGGAATTAAGAGAAGC	p.(Glu746_Ala750del)	19/28	rs121913421	inframe deletion	1997	1292	62.34%	64.16%	0.91	1.44
	TP53	c.673-2A>G	*TP53* null (frameshift)		rs1555525585	splice acceptor	15709	13794	99.71%	99.78%	0.04	0.04
**H2347**	*NRAS*	c.182A>G	p.(Gln61Arg)	3/7	rs11554290	missense	9000	7429	99.93%	99.89%	0.02	0.02

Abbreviations: *EGFR* – Epidermal Growth Factor Receptor; *NRAS – NRAS Proto-Oncogene, GTPase; TP53* – Tumor protein p53. Reference transcripts: NM_005228.5 for *EGFR*, NM_002524.5 for NRAS, and NM_000546.6 for *TP53* gene.

Table S3. The summary of the genetic variants identified in clinical samples from NSCLC patients using 'cobas' diagnostic qPCR system (Roche Diagnostics GmbH, Mannheim, Germany), and TST15 panel and MiSeq platform (Illumina, Inc., San Diego, CA, USA) for targeted NGS.

Sample No.	Age	Sex	Histology	Specimen type	Percen-tage of cancer cells	Gene	cobas qPCR results	NGS results								Agreement between assays for *EGFR* [Y/N]
								HGVSc	HGVSp	Exon	dbSNP	Variant type	Clinical significance (ClinVar)	Total Read Depth	Variant Read Frequency	
**1**	83	M	ADC	FFPET	60%	*EGFR*	G719x	c.2155G>A	p.(Gly719Ser)	18/28	rs28929495	missense	drug response	219705	0.0825	Y
						TP53	-	c.572_574delinsTTT	p.(Pro191_Gln192delins LeuTer)	6/11		frameshift indel	pathogenic	4503	0.4031	
**2**	63	M	ADC	FFPET	15%	*EGFR*	G719x	c.2155G>A	p.(Gly719Ser)	18/28	rs28929495	missense	drug response	47050	0.4376	Y
						EGFR	S768I	c.2303G>T	p.(Ser768Ile)	20/28	rs121913465	missense	pathogenic	26369	0.3423	Y
						TP53	-	c.96+1G>T			rs1131691003	splice donor	pathogenic	7394	0.2114	
**3**	79	F	ADC	FFPET	60%	*EGFR*	G719x	c.2156G>C	p.(Gly719Ala)	18/28	rs121913428	missense	drug response	33917	0.2941	Y
						TP53	-	c.738G>A	p.(Met246Ile)	7/11	rs1019340046	missense	pathogenic	15766	0.0445	
**4**	61	M	ADC	FFPET	15%	*EGFR*	G719x	c.2156G>C	p.(Gly719Ala)	18/28	rs121913428	missense	drug response	32407	0.1939	Y
						EGFR	S768I	c.2303G>T	p.(Ser768Ile)	20/28	rs121913465	missense	pathogenic	12654	0.1282	Y
**5**	75	F	ADC	FFPET	25%	*EGFR*	G719x	c.2156G>C	p.(Gly719Ala)	18/28	rs121913428	missense	drug response	9741	0.2157	Y
						TP53	-	c.734G>T	p.(Gly245Val)	7/11	rs121912656	missense	pathogenic	2282	0.3352	
**6**	62	F	ADC	FFPET	30%	*EGFR*	G719x	c.2156G>C	p.(Gly719Ala)	18/28	rs121913428	missense	drug response	13263	0.1890	Y
							L861Q	c.2582T>A	p.(Leu861Gln)	21/28	rs121913444	missense	drug response	387	0.1344	Y
						TP53	-	c.1045G>T	p.(Glu349Ter)	10/11	rs1567541975	missense	pathogenic	4520	0.1990	
**7**	78	M	ADC	FFPET	40%	*EGFR*	ex19del	c.2217_2234dup	p.(Ile740_Lys745dup)	19/28	rs397517090	inframe insertion	uncertain significance (VUS)	3749	0.7391	N
						TP53	-	c.659A>G	p.(Tyr220Cys)	6/11	rs121912666	missense	pathogenic	2879	0.3013	
**8**	77	F	ADC	FFPET	70%	*EGFR*	ex19del	c.2235_2249del	p.(Glu746_Ala750del)	19/28	rs121913421	inframe deletion	drug response	7054	0.6711	Y
						PIK3CA	-	c.1633G>A	p.(Glu545Lys)	10/21	rs104886003	missense	likely pathogenic	55401	0.1657	
**9**	72	F	ADC	FFPET	50%	*EGFR*	ex19del	c.2235_2249del	p.(Glu746_Ala750del)	19/28	rs121913421	inframe deletion	drug response	12705	0.6969	Y
						TP53	-	c.107dup	p.(Ser37ValfsTer6)	4/11		frameshift indel	pathogenic	6649	0.3856	
**10**	68	F	ADC	FFPET	70%	*EGFR*	ex19del	c.2235_2249del	p.(Glu746_Ala750del)	19/28	rs121913421	inframe deletion	drug response	3693	0.2564	Y
**11**	70	F	ADC	FFPET	40%	*EGFR*	ex19del	c.2235_2249del	p.(Glu746_Ala750del)	19/28	rs121913421	inframe deletion	drug response	17067	0.1349	Y
**12**	59	F	ADC	FFPET	60%	*EGFR*	ex19del	c.2235_2252delinsAAT	p.(Glu746_Thr751delinsIle)	19/28		indel	drug response	8310	0.3579	Y

						PIK3CA	-	c.1624G>A	p.(Glu542Lys)	10/21	rs121913273	missense	pathogenic	57959	0.1356	
						TP53	-	c.641A>G	p.(His214Arg)	6/11	rs1057519992	missense	likely pathogenic	4320	0.3307	
**13**	59	F	NOS	smear	90%	*EGFR*	ex19del	c.2236_2250del	p.(Glu746_Ala750del)	19/28	rs727504233	inframe deletion	drug response	6540	0.8402	Y
						TP53	-	c.823T>C	p.(Cys275Arg)	8/11	rs1057519983	missense	likely pathogenic	5313	0.7519	
**14**	47	M	ADC	FFPET	50%	*EGFR*	ex19del	c.2236_2250del	p.(Glu746_Ala750del)	19/28	rs727504233	inframe deletion	drug response	1612	0.4249	Y
							ex20ins	n/d	n/d							N
								c.2305G>A	p.(Val769Met)	20/28	rs147149347	missense	uncertain significance (VUS)	10732	0.0246	
						PIK3CA	-	c.1633G>A	p.(Glu545Lys)	10/21	rs104886003	missense	pathogenic	202	0.4752	
						TP53	-	c.920-1G>A			rs587781702	splice acceptor	pathogenic	1190	0.0623	
							-	c.548C>G	p.(Ser183Ter)	5/11	rs1555525970	nonsense	pathogenic	17583	0.1339	
**15**	68	M	NOS	smear	100%	*EGFR*	ex19del	c.2237_2255delAATTAAGAGAAGCAACATCinsT	p.(Glu746_Ser752delinsVal)	19/28	rs727504258	indel	drug response	3689	0.1149	Y
**16**	71	M	ADC	FFPET	70%	*EGFR*	ex19del	c.2237_2255delinsT	p.(Glu746_Ser752delinsVal)	19/28	rs727504258	indel	drug response	7344	0.3361	Y
						TP53	-	c.756_773del	p.(Thr253_Glu258del)	7/11		inframe deletion		14765	0.3134	
**17**	62	F	ADC	FFPET	30%	*EGFR*	ex19del	c.2239_2248delTTAAGAGAAGinsC	p.(Leu747_Ala750delinsPro)	19/28	rs727504278	indel	drug response	7950	0.3512	Y
**18**	74	F	ADC	FFPET	70%	*EGFR*	ex19del	n/d	n/d							N
							ex20ins	n/d	n/d							N
**19**	60	F	ADC	FFPET	40%	*EGFR*	n/d	c.2303G>T	p.(Ser768Ile)	20/28	rs121913465	missense	pathogenic	920	0.0913	N
**20**	82	M	ADC	FFPET	40%	*EGFR*	ex20ins	c.2300_2308dup	p.(Ala767_Val769dup)	20/28	rs727504263	inframe insertion	drug response	13704	0.3287	Y
**21**	82	M	ADC	FFPET	40%	*EGFR*	ex20ins	c.2300_2308dup	p.(Ala767_Val769dup)	20/28	rs727504263	inframe insertion	pathogenic	1064	0.2538	Y
						TP53	-	c.351dup	p.(Thr118AspfsTer31)	4/11		frameshift indel	pathogenic	1362	0.2217	
**22**	82	M	ADC	FFPET	100%	*EGFR*	ex20ins	c.2300_2308dup	p.(Ala767_Val769dup)	20/28	rs727504263	inframe insertion	pathogenic	1915	0.5937	Y
						TP53	-	c.536A>G	p.(His179Arg)	5/11	rs1057519991	missense	pathogenic	1067	0.9230	
**23**	69	F	ADC	FFPET	20%	*EGFR*	ex20ins	c.2300_2308dup	p.(Ala767_Val769dup)	20/28	rs727504263	inframe insertion	pathogenic	1697	0.1084	Y
**24**	71	F	NOS	FFPET	80%	*EGFR*	ex20ins	n/d	n/d							N
						BRAF	-	c.1799T>A	p.(Val600Glu)	15/18	rs113488022	missense	drug response	14481	0.4949	
**25**	53	F	ADC	FFPET	90%	*EGFR*	ex20ins	c.2300_2308dup	p.(Ala767_Val769dup)	20/28	rs727504263	inframe insertion	pathogenic	6978	0.5235	Y
						TP53	-	c.714T>G	p.(Cys238Trp)	7/11	rs193920789	missense	pathogenic/likely pathogenic	3705	0.4156	

**26**	59	F	ADC	FFPET	90%	*EGFR*	ex20ins	c.2300_2308dup	p.(Ala767_Val769dup)	20/28	rs727504263	inframe insertion	pathogenic	29827	0.8325	Y
**27**	66	F	ADC	FFPET	50%	*EGFR*	ex20ins	c.2300_2308dup	p.(Ala767_Val769dup)	20/28	rs727504263	inframe insertion	pathogenic	6377	0.4836	Y
**28**	58	F	ADC	FFPET	70%	*EGFR*	ex20ins	c.2303_2311dup	p.(Ser768_Asp770dup)	20/28	rs397517109	inframe insertion	pathogenic	19352	0.2723	Y
**29**	48	F	ADC	FFPET	70%	*EGFR*	ex20ins	c.2303_2311dup	p.(Ser768_Asp770dup)	20/28	rs397517109	inframe insertion	pathogenic	23848	0.8227	Y
**30**	72	F	ADC	FFPET	40%	*EGFR*	ex20ins	c.2303_2311dup	p.(Ser768_Asp770dup)	20/28	rs397517109	inframe insertion	pathogenic	3575	0.1695	Y
**31**	58	F	ADC	FFPET	60%	*EGFR*	ex20ins	c.2308_2309insGCAGCGTGG	p.(Val769_Asp770insGlySerVal	20/28		inframe insertion	pathogenic	9670	0.2477	Y
**32**	82	F	ADC	FFPET	50%	*EGFR*	S768I	c.2308_2309insGGGTCGTGG	p.(Val769_Asp770insGlyValVal)	20/28		inframe insertion	pathogenic	6940	0.2402	N
						TP53	-	c.722C>A	p.(Ser241Tyr)	7/11	rs28934573	missense	likely pathogenic	15311	0.3271	
**33**	71	F	ADC	FFPET	20%	*EGFR*	ex20ins	c.2311_2325dup	p.(Asn771_Cys775dup)	20/28		inframe insertion	pathogenic	4330	0.1674	Y
**34**	75	F	ADC	FFPET	80%	*EGFR*	ex20ins	c.2317_2319dup	p.(His773dup)	20/28	rs1554350381	inframe insertion	pathogenic	5922	0.4124	Y
**35**	53	F	ADC	FFPET	50%	*EGFR*	ex20ins	c.2317_2319dup	p.(His773dup)	20/28	rs1554350381	inframe insertion	pathogenic	30661	0.9207	Y
						TP53	-	c.733G>A	p.(Gly245Ser)	7/11	rs28934575	missense	pathogenic	3473	0.2719	
**36**	77	F	ADC	FFPET	70%	*EGFR*	ex20ins	c.2317_2319dup	p.(His773dup)	20/28	rs1554350381	inframe insertion	pathogenic	3684	0.3420	Y
**37**	48	F	ADC	FFPET	40%	*EGFR*	ex20ins	c.2317_2319dup	p.(His773dup)	20/28	rs1554350381	inframe insertion	pathogenic	5997	0.1161	Y
							-	c.2161G>A	p.(Gly721Ser)	18/28		missense	pathogenic	9186	0.0289	
						TP53	-	c.569del	p.(Pro190LeufsTer57)	6/11	rs1597368891	frameshift indel	pathogenic	3128	0.1186	
**38**	85	F	ADC	FFPET	50%	*EGFR*	ex20ins	c.2317_2319dup	p.(His773dup)	20/28	rs1554350381	inframe insertion	pathogenic	2670	0.1416	Y
**39**	82	M	ADC	FFPET	50%	*EGFR*	ex20ins	n/d	n/d							N
**40**	76	M	ADC	FFPET	60%	*EGFR*	ex20ins	n/d	n/d							N
						KRAS	-	c.34G>T	p.(Gly12Cys)	2/6	rs121913530	missense	pathogenic	3428	0.1826	
**41**	66	M	NOS	smear	10%	*EGFR*	L858R	c.2573T>G	p.(Leu858Arg)	21/28	rs121434568	missense	drug response	2619	0.0244	Y
						TP53	-	c.743G>A	p.(Arg248Gln)	7/11	rs11540652	missense	pathogenic	51873	0.0807	
**42**	72	M	ADC	FFPET	50%	*EGFR*	L858R	c.2573T>G	p.(Leu858Arg)	21/28	rs121434568	missense	drug response	1588	0.2062	Y
						EGFR	T790M	c.2369C>T	p.(Thr790Met)	20/28	rs121434569	missense	protective	17414	0.2048	Y
**43**	68	F	LCNEC	FFPET	50%	*EGFR*	L858R	c.2573T>G	p.(Leu858Arg)	21/28	rs121434568	missense	drug response	4697	0.9282	Y
**44**	80	F	ADC	FFPET	35%	*EGFR*	L858R	c.2573T>G	p.(Leu858Arg)	21/28	rs121434568	missense	drug response	2079	0.1785	Y
						TP53	-	c.321C>A	p.(Tyr107Ter)	4/11	rs770776262	nonsense	pathogenic	22664	0.2088	

**45**	83	F	ADC	FFPET	40%	*EGFR*	L858R	c.2573T>G	p.(Leu858Arg)	21/28	rs121434568	missense	drug response	710	0.2676	Y
							-	c.2327G>A	p.(Arg776His)	20/28	rs483352806	missense	likely pathogenic	10900	0.2751	
						TP53		c.154C>T	p.(Gln52Ter)	4/11		nonsense	pathogenic	13368	0.2041	
**46**	50	F	ADC	FFPET	20%	*EGFR*	L858R	c.2573T>G	p.(Leu858Arg)	21/28	rs121434568	missense	drug response	1608	0.1244	Y
						EGFR	S768I	c.2303G>T	p.(Ser768Ile)	20/28	rs121913465	missense	pathogenic	14373	0.2094	Y
**47**	69	F	ADC	FFPET	30%	*EGFR*	L858R	c.2573T>G	p.(Leu858Arg)	21/28	rs121434568	missense	drug response	540	0.2778	Y
						EGFR	S768I	c.2303G>T	p.(Ser768Ile)	20/28	rs121913465	missense	pathogenic	8393	0.2740	Y
						TP53	-	c.733G>T	p.(Gly245Cys)	7/11	rs28934575	missense	pathogenic	15370	0.1829	
**48**	71	M	ADC	FFPET	25%	*EGFR*	L858R	c.2573T>G	p.(Leu858Arg)	21/28	rs121434568	missense	drug response	1312	0.8716	Y
							ex20ins	n/d	n/d							N
**49**	77	M	ADC	smear	<10%	*EGFR*	n/d	n/d	n/d							Y
**50**	53	M	ADC	FFPET	50%	*EGFR*	n/d	n/d	n/d							Y
						TP53	-	c.747G>T	p.(Arg249Ser)	7/11	rs28934571	missense	pathogenic	11495	0.1692	
**51**	67	F	ADC	FFPET	15%	*EGFR*	n/d	n/d	n/d							Y
						KRAS	-	c.34G>A	p.(Gly12Ser)	2/6	rs121913530	missense	pathogenic	2589	0.2965	
						TP53	-	c.736A>G	p.(Met246Val)	7/11	rs483352695	missense	pathogenic	8905	0.1917	
**52**	71	M	ADC	smear	100%	*EGFR*	n/d	n/d	n/d							Y
						KRAS	-	c.35G>A	p.(Gly12Asp)	2/6	rs121913529	missense	pathogenic	2422	0.2155	
**53**	56	M	ADC	FFPET	60%	*EGFR*	n/d	n/d	n/d							Y
						KRAS	-	c.34G>T	p.(Gly12Cys)	2/6	rs121913530	missense	pathogenic	1078	0.2407	
						TP53	-	c.734G>T	p.(Gly245Val)	7/11	rs121912656	missense	pathogenic	3107	0.1972	
**54**	82	F	ADC	FFPET	70%	*EGFR*	n/d	n/d	n/d							Y
						BRAF	-	c.1799T>A	p.(Val600Glu)	15/18	rs113488022	missense	drug response	885	0.1266	
**55**	82	F	NOS	smear	80%	*EGFR*	n/d	n/d	n/d							Y
						TP53	-	c.880G>T	p.(Glu294Ter)	8/11	rs1057520607	nonsense	pathogenic	4263	0.1167	
							-	c.592del	p.(Glu198LysfsTer49)	6/11	rs1131691035	frameshift indel	pathogenic	9095	0.1727	
**56**	65	F	NOS	smear	90%	*EGFR*	n/d	n/d	n/d							Y
**57**	68	M	ADC	FFPET	100%	*EGFR*	n/d	n/d	n/d							Y
						TP53	-	c.823del	p.(Cys275ValfsTer70)	8/11		frameshift indel	pathogenic	7947	0.1150	

**58**	64	M	ADC	smear	80%	*EGFR*	n/d	n/d	n/d							Y
**59**	64	M	LCNEC	FFPET	10%	*EGFR*	n/d	n/d	n/d							Y
						TP53	-	c.535C>T	p.(His179Tyr)	5/11	rs587780070	missense	pathogenic	12603	0.2186	

Abbreviations: ADC - Adenocarcinoma; LCNEC - Large Cell Neuroendocrine Carcinoma of the Lung; NOS - Not otherwise specified; FFPET - Formalin-Fixed Paraffin-Embedded Tissue; *BRAF* – B-Raf Proto-Oncogene, Serine/Threonine Kinase; *EGFR* – Epidermal Growth Factor Receptor; *KRAS* – Protein V-Ki-ras2 Kirsten rat sarcoma viral oncogene homolog; *PIK3CA* – Phosphatidylinositol-4,5-Bisphosphate 3-Kinase Catalytic Subunit Alpha; *TP53* – Tumor protein P53. Reference cDNA (protein) sequence: NM_004333.6 (NP_004324.2) for *BRAF*, NM_005228.5 (NP_005219.2) for *EGFR*, NM_004985.5 (NP_004976.2) for *KRAS*, NM_006218.4 (NP_006209.2) for *PIK3CA*, and NM_000546.6 (NP_000537.3) for *TP53* gene.

Table S4. The coverage in *EGFR*-negative samples evaluated by targeted NGS assay (including two samples where qPCR and NGS showed discrepancy for exon 20 insertion) for all the *EGFR* amplicons sequenced.

**Amplicon**	**Chromo-some**	**Start Position**	**End Position**	***EGFR* exon**	**Sample No. / Amplicon coverage in *EGFR*-negative samples (number of reads)**																
					**50**	**57**	**49**	**52**	**58**	**55**	**51**	**53**	**54**	**59**	**56**	**1450**	**48***	**39**	**18**	**14***	**24**
1EGFRxxE18TF030SR030.1	chr7	55241562	55241676	18	14404	19750	13594	6691	13824	6190	4844	5081	5742	2427	5639	3112	18303	19902	5603	2642	7719
2EGFRxxE18TF031SR031.1	chr7	55241664	55241778	18	36166	47461	34193	15588	36627	12183	14195	9165	8029	2627	13304	726	45596	62671	10598	3242	12281
1EGFRxxE19TF032SR032.1	chr7	55242413	55242517	19	5032	6590	6718	2566	4697	2244	2562	2737	1405	686	2038	1304	4808	4834	1242	1646	2126
2EGFRxxE20TF033SR033.1	chr7	55248972	55249100	20	30416	36174	18220	12945	33078	14686	10353	6288	14658	13714	8463	7858	23267	46248	8726	11029	7171
1EGFRxxE20TF034SR034.1	chr7	55249072	55249200	20	49100	79975	48179	32617	63013	25850	18504	20318	18433	11148	24004	9394	40226	60286	19254	4758	23773
1EGFRxxE21TF035SR035.1	chr7	55259485	55259630	21	1636	2305	1683	575	1005	502	512	233	231	72	528	257	1351	1658	204	42	619

* one of the two *EGFR* variants detected by NGS showed discrepancy with qPCR result

Table S5. The characteristics of the 'cobas' allele-specific real-time PCR-based diagnostic system (Roche Diagnostics GmbH, Mannheim, Germany) for *EGFR* mutation detection in tumor specimens.

Characteristics	cobas *EGFR* mutation detection platform
**Manufacturer**	**Roche Diagnostics GmbH, Mannheim, Germany**
**Dedicated cfDNA extraction kit**	**cobas cfDNA Sample Preparation Kit**
Number of samples processed per assay	24
DNA extraction technique	glass fiber-filter column
Sample processing	manual (centrifuge protocol)
IVD certificate	yes
**Dedicated kit for *EGFR* mutation detection in FFPE**	**cobas EGFR Mutation Test v2**
Allele-specific PCR amplification system	TaqMAMA
Number of samples analyzed per run	24
Number of mutated EGFR variants detected	42
Deletions in exon 19	29
p.T790M	1
p.L858R	2
p.L861Q	1
p.G719X (G719S, G719A, or G719C)	3
p.S768I	1
Insertions in exon 20	5
Positive Control mutant DNA included	yes
Parallel amplification of DNA from FFPE tissue and ctDNA from plasma	yes
UDG-based prevention of carryover contamination	yes
Limit of detection (LOD) by manufacturer	Depends on the variant; 1.3% (S768I) - 13.4% (Leu747_Pro753delinsSer)
IVD certificate	yes
**qPCR platform + software**	**cobas z480 Instrument + software version 2.2.0**
Automated data analysis and reporting	yes
Parallel analysis of *EGFR* mutations in FFPE tissue and plasma ctDNA	yes
IVD certificate	yes
**Diagnostic application**	**companion diagnostic test**
**CE mark**	**yes (September, 2015)**
**FDA approval**	**yes (June 1, 2016)**

Abbreviations: EGFR – epidermal growth factor receptor, cfDNA – cell-free DNA, IVD – in vitro diagnostic, TaqMAMA – fluorogenic 5' nuclease polymerase chain reaction (TaqMan) and the mismatch amplification mutation assay (MAMA), (allele-specific primers and probes; FFPE – formalin-fixed paraffin-embedded, UDG – uracil-DNA glycosylase, p.L858R – leucine to arginine substitution at position 858 in amino-acid sequence of EGFR; p.L861Q – leucine to glutamine substitution at position 861 in amino-acid sequence of EGFR; p.G719X – glycine to serine (G719S), alanine (G719A) or cysteine (G719C) substitution at position 719 in amino-acid sequence of EGFR; p.S768I – serine to isoleucine substitution at position 768 in amino-acid sequence of EGFR; p. – for a protein sequence, CE – *Conformité Européenne*, conformity with European health, safety, and environmental protection standards for products sold within the European Economic Area; FDA – Food and Drug Administration.

Table S6. The list of variants detected by the cobas EGFR mutation test v2 (Roche Diagnostics GmbH, Mannheim, Germany).

No.	Exon	*EGFR* Mutation Group	DNA sequence change (HGVSc)	Amino acid change (HGVSp)	COSMIC ID	dbSNP
1.	Exon 18	G719x *	c.2156G>C	p.Gly719Ala	6239	rs121913428
2.			c.2155G>A	p.Gly719Ser	6252	rs28929495
3.			c.2155G>T	p.Gly719Cys	6253	rs28929495
4.	Exon 19	Ex19Del *	c.2240_2251del	p.Leu747_Thr751delinsSer	6210	rs121913441
5.			c.2239_2247del	p.Leu747_Glu749del	6218	rs121913436
6.			c.2238_2255del	p.Glu746_Ser752delinsAsp	6220	rs121913423
7.			c.2235_2249del	p.Glu746_Ala750del	6223	rs121913421
8.			c.2236_2250del	p.Glu746_Ala750del	6225	rs727504233
9.			c.2239_2253del	p.Leu747_Thr751del	6254	rs121913442
10.			c.2239_2256del	p.Leu747_Ser752del	6255	rs121913440
11.			c.2237_2254del	p.Glu746_Ser752delinsAla	12367	rs121913422
12.			c.2240_2254del	p.Leu747_Thr751del	12369	rs121913442
13.			c.2240_2257del	p.Leu747_Pro753delinsSer	12370	rs121913438
14.			c.2239_2248delTTAAGAGAAGinsC	p.Leu747_Ala750delinsPro	12382	rs727504278
15.			c.2239_2251delinsC	p.Leu747_Thr751delinsPro	12383	rs397509368
16.			c.2237_2255delinsT	p.Glu746_Ser752delinsVal	12384	rs727504258
17.			c.2235_2255delinsAAT	p.Glu746_Ser752delinsIle	12385	
18.			c.2237_2252delinsT	p.Glu746_Thr751delinsVal	12386	
19.			c.2239_2258delinsCA	p.Leu747_Pro753delinsGln	12387	rs121913437
20.			c.2239_2256delinsCAA	p.Leu747_Ser752delinsGln	12403	
21.			c.2237_2253delinsTTGCT	p.Glu746_Thr751delinsValAla	12416	
22.			c.2238_2252delinsGCA	p.Leu747_Thr751delinsGln	12419	
23.			c.2238_2248delinsGC	p.Leu747_Ala750delinsPro	12422	rs121913435
24.			c.2237_2251del	p.Glu746_Thr751delinsAla	12678	rs121913425
25.			c.2236_2253del	p.Glu746_Thr751del	12728	rs121913426
26.			c.2235_2248delinsAATTC	p.Glu746_Ala750delinsIlePro	13550	rs727504281
27.			c.2235_2252delinsAAT	p.Glu746_Thr751delinsIle	13551	
28.			c.2235_2251delinsAATTC	p.Glu746_Thr751delinsIlePro	13552	rs727504332
29.			c.2253_2276del	p.Ser752_Ile759del	13556	rs727504232
30.			c.2237_2257delinsTCT	p.Glu746_Pro753delinsValSer	18427	rs727504282
31.			c.2238_2252del	p.Leu747_Thr751del	23571	rs121913442
32.			c.2233_2247del	p.Lys745_Glu749del	26038	rs2128954710
33.	Exon 20	S768I	c.2303G>T	p.Ser768Ile	6241	rs121913465
34.		T790M	c.2369C>T	p.Thr790Met	6240	rs121434569
35.		Ex20Ins *	c.2300_2308dup	p.Ala767_Val769dup	12376	rs727504263
36.			c.2317_2319dup	p.His773dup	12377	rs1554350381
37.			c.2310_2311insGGT	p.Asp770_Asn771insGly	12378	rs397517111
38.			c.2303_2311dup	p.Ser768_Asp770dup	13428	
39.			c.2309_2310delACinsCCAGCGTGGAT	p.Ala767_Val769dup	13558	rs2128958359
40.	Exon 21	L858R	c.2573T>G	p.Leu858Arg	6224	rs121434568
41.			c.2573_2574delTGinsGT	p.Leu858Arg	12429	rs1057519848
42.		L861Q	c.2582T>A	p.L861Q	6213	rs121913444

* the test detects the variants but does not differentiate them. Reference transcript for *EGFR*: NM_005228.5.

Table S7. The characteristics of non-small cell lung carcinoma (NSCLC) cell lines used to prepare the biological DNA reference material for the NGS assay evaluation.

**Cell line** (ATCC/ECACC ref. no.)	**Morphology**	**Cancer type**	**Explant source**	**Donor's ethnicity**	**Donor's gender**
**H2347** (ATCC, CRL-5942)	epithelial	NSCLC, adenocarcinoma	primary tumor	Caucasian	Female
**HCC4006** (ATCC, CRL-2871)	epithelial	NSCLC, adenocarcinoma	metastatic site	Caucasian	Male
**PC-9** (ECACC, 90071810)	epithelial	NSCLC, adenocarcinoma	primary tumor	Asian	Male
**H1975** (ATCC, CRL-5908)	epithelial	NSCLC, adenocarcinoma	primary tumor	unknown	Female
**H1650** (ATCC, CRL-5883)	epithelial	NSCLC, adenocarcinoma	metastatic site	Caucasian	Male

Abbreviations: ATCC - American Type Culture Collection; ECACC - European Collection of Authenticated Cell Cultures

Table S8. The list of the 15 genes and their regions covered by the TruSight Tumor 15 (TST15) sequencing panel (Illumina, Inc., San Diego, CA, USA).

AKT1	Exon 3* ; E17K	***GNA11***	Exon 5*; Q209L	***NRAS***	Exons 2* , 3* (partial), 4 Codons 12, 13, 59, 61, 117, 146
BRAF	Exon 15* (partial); V600E/K/R/M	***GNAQ***	Exon 5* (partial); Q209L	***PDGFRA***	Exons 12, 14, 18
EGFR	Focal Amplification, Exons 12* (partial), 18, 19, 20; G719A, G719X; Exon 21 (L858R), L861Q, S7681, T790M	***KIT***	Exons 8, 9, 10, 11, 13, 14, 17, 18	***PIK3CA***	Exons 9, 20
ERBB2	Focal Amplification, p.E770_A771insAYVM Exons 14* (partial), 17, 18, 19, 20* (partial), 21* (partial), 24, 26	***KRAS***	Exon 2* (partial), 3* (partial), 4	***RET***	Exon 16 (M918T)
FOXL2	Exon 1* (partial); C134W	***MET***	Focal Amplification	***TP53***	Full coding sequence

Abbreviations: *AKT1* – AKT Serine/Threonine Kinase 1; *BRAF* – B-Raf Proto-Oncogene, Serine/Threonine Kinase; *EGFR* – Epidermal Growth Factor Receptor; *ERBB2* – Erb-B2 Receptor Tyrosine Kinase; *FOXL2* – Forkhead Box L2; *GNA11* – G Protein Subunit Alpha 11; *GNAQ* – G Protein Subunit Alpha Q; *KIT* – KIT Proto-Oncogene, Receptor Tyrosine Kinase; *KRAS* – Protein V-Ki-ras2 Kirsten rat sarcoma viral oncogene homolog; *MET* - MET Proto-Oncogene, Receptor Tyrosine Kinase; *NRAS* – NRAS Proto-Oncogene, GTPase; *PDGFRA* – Platelet Derived Growth Factor Receptor Alpha; *PIK3CA* – Phosphatidylinositol-4,5-Bisphosphate 3-Kinase Catalytic Subunit Alpha; *RET* – Ret Proto-Oncogene; *TP53* – Tumor protein P53. *Coverage of these exons is only partial and targets specific hotspot.

The BED files for the TruSight Tumor 15 assay (Illumina) are available at the manufacturer's website: https://support.illumina.com/downloads/trusight-tumor-15-product-files.html

Certificate of Analysis

For Research Use Only
Not for Use in Diagnostic Procedures

Product Description:	Seraseq™ Tri-Level Tumor Mutation DNA Mix v2, RUO		
Material Number:	0710-0097	Batch Number:	10621387
Volume:	25 µL	Concentration:	25 ng/µL
Date of Manufacture:	06-APR-2022	Expiration Date:	06-APR-2024
Test Method for Concentration:	Qubit dsDNA BR Assay		
Average Concentration (ng/µL):	23.3 ng/µL		
Test Method for Variant Allele Frequency:	In house validated, allele specific, digital PCR assays		

4% Tier	
**Mutation**	**Avg. dPCR Allele Frequency**
BRAF COSM476	4.5
EGFR COSM12378	3.3
EGFR COSM6225	3.5
EGFR COSM6240	3.3
FGFR3 COSM715	3.7
FLT3 COSM783	3.9
FOXL2 COSM33661	4.5
IDH1 COSM28747	4.0
PDGFRA COSM28053	3.1
PIK3CA COSM775	4.0
PIK3CA COSM12464	3.8
RET COSM965	3.8
TP53 COSM18610	Not Assayed

10% Tier	
**Mutation**	**Average dPCR Allele Frequency**
AKT1 COSM33765	9.6
APC COSM13127	10.6
APC COSM18561	10.1
ATM COSM21924	9.6
ERBB2 COSM682	8.1
GNA11 COSM52969	9.4
GNAQ COSM28758	10.3
KIT COSM1314	10.4
MPL COSM18918	11.7
NCOA4-RET	10.7
PDGFRA COSM736	10.9
PIK3CA COSM763	9.7
SMAD4 COSM14105	10.3
TPR-ALK	10.7

7% Tier	
**Mutation**	**Average dPCR Allele Frequency**
CTNNB COSM5664	7.4
EGFR COSM6224	7.3
GNAS COSM27887	6.8
JAK2 COSM12600	8.4
KRAS COSM521	7.7
NPM1 COSM17559	6.1
NRAS COSM584	7.3
PTEN COSM4986	7.0
PTEN COSM5809	7.0
TP53 COSM10648	7.2
TP53 COSM6530	6.8
TP53 COSM10662	7.3
TP53 COSM10660	7.1

Approval:

Prepared By [signature] Date 16-MAY-2022

QA Verified By [signature] Date 05/16/2022

Figure S1. The certificate of analysis for the Seraseq Tri-Level Tumor Mutation DNA Mix v2 used in the study as the biosynthetic DNA reference material for NGS performance evaluation.
